# Effect of different artificial shrinkage methods, when applied before blastocyst vitrification, on perinatal outcomes

**DOI:** 10.1186/s12958-017-0252-7

**Published:** 2017-04-26

**Authors:** Caizhu Wang, Guixue Feng, Bo Zhang, Hong Zhou, Jinhui Shu, Ruoyun Lin, Huanhua Chen, Zhulian Wu

**Affiliations:** Center of Reproductive Medicine, Guangxi Maternal and Child Health Hospital, Nanning, Guangxi 530003 China

**Keywords:** Blastocyst, Vitrification, Artificial shrinkage, Clinical outcome, Perinatal outcome

## Abstract

**Background:**

In recent years, single blastocyst transfer combined with vitrification has been applied widely, which can maximize the cumulative pregnancy rate in per oocyte retrieval cycles and minimize the multiple pregnancy rate. Thus, the guarantee for these is the effectiveness of vitrified blastocyst. Studies has shown that AS of the blastocoel cavity prior to vitrification can reduce injuries, increase the thawed blastocyst survival rate and implantation rate. Several AS methods have been established. However, only a few studies have compared the effectiveness and safety of these AS methods. In this study, we aimed to compare the clinical outcomes and neonatal outcomes in FET cycles with single blastocyst that were artificially shrunk before vitrification by either LAS or MNAS method.

**Methods:**

A retrospective comparative study of FET cycles in infertile patients which were at our clinic between January 2013 and December 2014. These FET cycles were divided into two groups by the shrinking methods used before vitrification and the clinical and neonatal outcomes were assessed.

**Results:**

There were no statistically differences in blastocyst survival rates (95.40% vs 94.05%, *P* > 0.05) between the LAS and MNAS groups. However, compared with MNAS, LAS improved the warmed blastocyst implantation/clinical pregnancy rate (60.82% vs 54.37%, *P* < 0.05), live birth rate (50.43% vs 45.22%, *P* < 0.05) and also increased the monozygotic twin rate (4.07% vs 1.73%, *P* < 0.05). There were no differences in the average gestational weeks (38.83 ± 1.57 vs 38.74 ± 1.75), premature birth rate (0.30% vs 0.49%), average birth weight (3217.89 ± 489.98 g vs 3150.88 ± 524.03 g), low birth weight rate (5.60% vs 8.63%) and malformation rate (0.59% vs 0.48%) (*P* > 0.05).

**Conclusions:**

No significant differences in neonatal outcomes were observed, while in clinical outcomes, LAS improved the warmed blastocyst implantation/clinical pregnancy rate and live birth rate markedly, there was also an increased risk of monozygotic twin pregnancies.

## Background

The dilemma of how to maintain a high pregnancy rate and reduce the multiple pregnancy rate has become one of the most concerned issues in assisted reproductive technology (ART). Many studies confirmed that blastocyst transfer is highly favored because of its unique advantages such as high implantation rates and the ability to maintain endometrial synchronization during the process [1–3]. Therefore, blastocyst transfer has been applied widely [4]. Since the first birth after vitrification of human blastocyst was reported [5], attention has focused on vitrification and conventional slow-freezing methods were gradually replaced by vitrification.

Single blastocyst transfer combined with vitrification can maximize the cumulative pregnancy rate in per oocyte retrieval cycles and minimize the multiple pregnancy rate [6]. Thus, the effectiveness of vitrification is critical. However, during this process the blastocoel cavity is filled with large amounts of fluid, which may cause intracellular ice crystal formation and potentially lethal damage of the embryo during cooling by influencing the dehydration shrinkage rate. Studies has shown that artificial shrinkage (AS) of the blastocoel cavity prior to vitrification can reduce injuries due to chilling and increase the rates of thawed blastocyst survival and improve the implantation and pregnancy rate remarkably [7–10]. Therefore AS has been used widely prior to vitrification. Several AS methods have been established, such as micro-needle AS (MNAS), micro-pipetting AS, 29-gauge needle and laser pulse AS (LAS), which can significantly improve the thawed blastocyst survival rate and clinical pregnancy rate [10–12]. However, only a few studies focused on the comparison of these AS methods. 

In this study, we compared the clinical outcomes and neonatal outcomes in frozen embryo transfer (FET) cycles with single blastocyst that were artificially shrunk before vitrification by either LAS or MNAS.

## Methods

### Patients and FET data

Patients were recruited from January 2013 to December 2014, who underwent single frozen blastocyst transfer. Inclusion criteria: the warmed blastocysts graded to at least B for both trophectoderm and inner cell mass (ICM), the blastocoele expanded at least to stage III, first or second FET cycle and a single blastocyst transferred. The grade of blastocyst was conducted according to the Gardner’s grading system [13]. Of the 1552 FET cycles containing 1638 blastocysts used in this study, 790 blastocysts were assigned to the MNAS group (vitrification after MNAS) and 848 blastocysts were from the LAS group (vitrification after LAS).

### Methods

#### Embryo culture and blastocyst formation

Quinn’s medium was used for embryo culture. The embryo transfer strategy was performed with informed consent according to day 3 embryo quality and patients’ conditions. The day of oocyte retrieval was considered as day 0. If patients chosen D3 cleavage embryo transfer, then supernumerary embryos were cultured to day 5 or 6. If patients agreed to performed blastocyst culture, all embryos would be transferred into blastocyst culture medium. After day 5 of embryo transfer, all the remaining blastocysts graded at least BC or CB that reached stage 3 or more on day 5 or 6 according to the Gardner’s grading system [13] were vitrified.

#### Artificial shrinkage of the blastocyst

Artificial shrinkage of the blastocoel was induced by applying MNAS or LAS at room temperature (24 ± 2 °C).

##### Micro-needle AS

A holding needle was used to stabilize the blastocyst, with the ICM at the direction of 12 or 6 o’clock. An injection needle was used to pierce through the expanded blastocyst from the area with the least number of trophoblast cells. The fluid in the blastocoel cavity flowed out during the process of needle injection aspiration, resulting in rapid shrinkage of the blastocyst.

##### Laser pulse AS

Using the RI Saturn Laser System (England), one single laser shot (409 μs) was delivered to one of the trophoblast cells, while the ICM was positioned far from the shooting spot. Repeated micro-pipetting with a hand-drawn Pasteur pipette helps to achieve complete shrinkage of the blastocyst.

#### Vitrification and thawing of blastocyst

Vitrification and warming were performed according to the methods established by our clinic and described previously [14].

##### Vitrification

The blastocysts were placed into 200 μL HEPES-buffered culture medium (Quinn’s-1023, SAGE, USA) supplemented with 20% human serum albumin (HSA, SAGE, USA) and rinsed for 30s. Then this was moved into a 200 μL droplet of equilibration solution containing 10% (*v/v*) ethylene glycol (American SIGMA) and 10% (*v/v*) DMSO (American SIGMA) for 1 min incubation. After that, the blastocyst was transferred to a 200 μL droplet of vitrification solution containing 20% (*v/v*) ethylene glycol, 20% (*v/v*) DMSO and 0.3 mol/L sucrose (American SIGMA) for 30s, and then a micro-droplet (<0.5 μL) with the blastocyst was sucked into the glass micro-pipette by a siphoning effect, and the micro-pipette was plunged into liquid nitrogen immediately for cryopreservation.

##### Thawing

On the day of transfer in FET cycles, blastocysts were warmed. For warming, a petri dish containing 200 μL droplets with four different thawing solutions (TS1 0.6 mol/L sucrose in HEPES-buffered media supplemented with 20% HAS, TS2 0.5 molL/L sucrose in HEPES-buffered media supplemented with 20% HAS, TS3 0.25 mol/L sucrose in HEPES-buffered media supplemented with 20% HAS and TS4 HEPES-buffered media supplemented with 20% HSA) were made and then kept at 37 °C. For warming, the capillary end of the glass micro-pipette with blastocyst was placed into the TS1 quickly, then the blastocyst was released from the capillary and incubated for 2 min. Then, the blastocyst was incubated in TS2 for 3 min, in TS3 for 3 min and in TS4 for 5 min. After warming, the blastocyst was transferred to the blastocyst culture medium and cultured for 2–4 h in the incubator to assess its morphological survival. If the blastocyst was damaged severely with more than half cells showing signs of damage or the blastocyst was regressing with no signs of re-expansion [15], an extra one was warmed immediately if the patient had another frozen blastocyst in reserve. Otherwise, the FET cycle was be cancelled.

#### Quality evaluation of warmed blastocyst

The quality of warmed blastocyst was assessed 2–4 h post-culture on the inverted microscope by two independent embryologists according to the Gardner’s criteria [13].

#### Transfer of warmed blastocyst

The common modality for FET were the natural cycles or hormone replacement cycles for endometrial preparation. Blastocyst transfer was performed under ultrasound guidance using an embryo transfer catheter on day 6 after ovulation or progesterone injection. After transplantation, intramuscular injection of progesterone was administered as a routine scheme for luteal support.

### Follow-up and evaluation index

On the 14th day post-transfer, patients whose serum HCG were positive were identified as positive for a biochemical pregnancy. On the 28th day post-transfer, the gestational sac was monitored by trans-vaginal ultrasound to confirm the clinical pregnancy. In mid-trimester pregnancy and third trimester pregnancy, details of the patients’ ongoing pregnancy were recorded by a follow-up phone call. Live birth rate was defined as live birth delivery cycles divided by transfer cycles. Less than 32 gestational weeks was defined as premature birth and 28 weeks as very premature birth. Birth weight lower than 2,500 g was defined as low birth weight and birth weight lower than 1,500 g was defined as very low birth weight.

### Statistical analysis

Statistical analysis was completed with the SPSS 13.0 package. Data are summarized with the use of means ± SD. The means were compared by analysis of variance (ANOVA) and proportional data were compared in *χ*2 analysis. *P* < 0.05 was considered statistically significant.

## Results

### Characteristics of patients

As shown in Table 1 the mean age of patients, average duration of infertility, mean number of cycles, frequency of abortion, BMI, basic FSH, basic LH, endometrial preparation and average endometrial thickness on the day of embryo transfer were not statistically significant between the MNAS and LAS groups (*P* > 0.05). There was no difference in average number of blastocysts that were frozen and the mean number of embryos produced before blastocyst formation (*P* > 0.05).

**Table 1:** Basic clinical data from subjects in this study

	Micro-needle AS	Laser pulse AS	*P*
FET cycles (n)	743	809	----
Age (y)	30.75 ± 3.49	30.53 ± 3.38	0.203
Infertility duration (y)	3.65 ± 2.74	3.87 ± 2.88	0.120
Times of abortion	0.72 ± 0.91	0.71 ± 0.98	0.933
BMI (kg/m^2^)	20.89 ± 2.43	21.13 ± 2.50	0.056
Endometrial thickness (mm)	9.44 ± 1.77	9.47 ± 1.77	0.765
Basal FSH (IU/L)	6.95 ± 1.78	6.81 ± 1.68	0.111
Basal LH (IU/L)	5.81 ± 3.12	5.82 ± 3.67	0.967
Percentage of natural cycle	66.08 (491/743)	61.93 (501/809)	0.089
Mean number of frozen blastocysts	5.09 ± 3.30	5.18 ± 3.40	0.204
Mean number of embryos used for culture blastocyst	11.72 ± 6.09	11.99 ± 6.13	0.428
Mean number of cycles	1.19 ± 0.39	1.17 ± 0.37	0.0714
Mean number of collected oocytes	16.83 ± 7.21	17.47 ± 7.59	0.077

### Comparison of clinical outcome

In the LAS group, there were 809 FET cycles containing 848 warmed blastocysts, of which 809 survived (survival rate was 95.40%). In the MNAS group, there were 743 FET cycles including 790 blastocysts, of which 743 survived (survival rate was 94.05%). No difference in this rate was detected(95.40% vs 94.05%, *P* > 0.05). The implantation rates/clinical pregnancy rates (60.82% vs 54.37%) and live birth rates (50.43% vs 45.22%) in the LAS group were significantly higher than the MNAS group (*P* < 0.05). However, the incidence rates of monozygotic twins in the LAS group were also significantly higher than the MNAS group (4.07% vs 1.73%, *P* < 0.05). There was no statistically significant in the abortion rates and ectopic pregnancy rates between these two groups (*P* > 0.05; Table 2).

**Table 2:** Analysis of clinical outcome

	Micro-needle AS	Laser pulse AS	*P*
FET cycles (n)	743	809	
Blastocyst survival rate (%)	94.05 (743/790)	95.40 (809/848)	0.221
Mean number of blastocysts thawed	1.07 ± 0.26	1.05 ± 0.21	0.096
Implantation/pregnancy rate (%, n)	54.37 (404/743)	60.82 (492/809)	0.010
Monozygotic twin rate (%, n)	1.73 (7/404)	4.07 (20/492)	0.042
Ectopic pregnancy rate (%, n)	0.74 (3/404)	1.22 (6/492)	0.707
Abortion rate (%, n)	15.84 (64/404)	15.04 (74/492)	0.741
Delivery cycles	337	412	
Live birth cycles	336	408	
Stillbirth cycles	1	4	
Live birth rate (%, n)	45.22 (336/743)	50.43 (408/809)	0.040

### Comparison of neonatal outcome

As shown in Table 3 a total of 339 babies were born in 336 live birth cycles in the MNAS group, and 417 babies were born in 408 live birth cycles in the LAS group. There were no differences with respect to the average gestational weeks (38.83 ± 1.57w vs 38.74 ± 1.75w), mean birth weight (3217.89 ± 489.98 g vs 3150.88 ± 524.03 g), premature birth rates (0.30% vs 0.49%), low weight rates (5.60% vs 8.63%) and very low weight rates (0.29% 0.72%) between the MNAS and LAS groups (*P* > 0.05). The malformation rates in the MNAS and LAS groups respectively were 0.59% and 0.48%, there was also no difference (*P* > 0.05).

**Table 3:** Analysis of neonatal outcome

	Micro-needle AS	Laser pulse AS	*P*
Live birth cycles	336	408	
Number of babies	339	417	
Mean gestational age (weeks)	38.83 ± 1.57	38.74 ± 1.75	0.479
Premature birth rate (%, n)	0.30 (1/336)	0.49 (2/408)	1.000
Very premature birth rate (%)	0	0	
Mean birth weight (g)	3217.89 ± 489.98	3150.88 ± 524.03	0.072
Low weight rate (%, n)	5.60 (19/339)	8.63 (36/417)	0.111
Very low weight rate (%, n)	0.29 (1/339)	0.72 (3/417)	0.767
Malformation rate (%, n)	0.59 (2/339)	0.48 (2/417)	1.000

## Discussion

Large amounts of fluid in blastocoelic cavity may influence the permeation of cryoprotectant during vitrification, which may cause intracellular ice crystal formation and result in adverse impact on blastocyst viability. AS can induce rapid collapse of the blastocoel and improve the warmed blastocyst survival rate. Many studies have confirmed that AS of expanded blastocyst prior to vitrification can remarkably increase the blastocyst survival rate, implantation rate and clinical pregnancy rate [7–11]. Therefore, AS has been widely applied in vitrification of the blastocyst. It was also found that AS pre-processing can also be applied prior to the fresh blastocyst transfer to improve the clinical outcome by selecting the most rapid re-expansion blastocyst [16]. So, it seems that AS does not cause damage to the blastocyst.

The AS methods commonly used currently are MNAS [7], micro-pipetting AS [8], LAS [9] and 29-gauge needle AS [12]. All these AS methods have been shown to improve blastocyst survival rate, implantation rate and pregnancy rate [7, 9, 11, 12, 17, 18]. However, only a few studies focused on the comparison of these AS methods when applied prior to vitrification. Therefore, we conducted a comprehensive study using 1552 FET cycles from January 2013 to December 2014 with detailed medical record and, intended to compare the influence of the MNAS and LAS methods when applied before blastocyst vitrification.

The results showed that the blastocyst survival rates were similar, but the blastocyst implantation rate and clinical pregnancy rate in the LAS group was significantly higher than the MNAS group. However, Mukaida et al. found that LAS and MNAS could achieve similar blastocyst survival rates, implantation rates and abortion rates [9]. Van Landuyt et al. conducted a prospective randomized controlled trial and showed that the LAS method improved blastocyst survival rate markedly, but failed to increase the implantation rate of post-warmed blastocyst [19]. These results were inconsistent with ours. It can be speculated that MNAS may cause more damage to the trophoderm, which plays an important role in implantation, relative to that caused by the LAS method. Desai found that there was a trend of a faster and higher re-expansion rate after LAS compared with MNAS [20]. Faster and higher re-expansion can be a strong predictor of clinical pregnancy outcome [21]. So, this may be a good explanation for the high implantation rate of post-warmed blastocyst artificial shrinkage by laser. In addition, in our hands artificial shrinkage by laser is a more time-saving procedure.

Currently, the safety aspects of AS remain controversial. Most of the published research data involve no more than clinical outcome parameters, with very little data regarding analysis on neonatal outcomes. A study showed that using the 29-gauge needle AS method resulted with a higher premature birth rate (40.00% vs. 21.15%, *P* < 0.05) when compared with an LAS group [22]. Levi-Setti et al., found that MNAS did not increase the abortion rate, premature birth rate and low birth weight rate [23]. Our results also showed similar neonatal outcomes in the LAS and MNAS groups. The premature birth rates and low birth weight rate in the MNAS and LAS groups were 0.3% vs 0.49% and 5.6% vs 8.63% respectively. The neonatal outcome parameters of premature birth rate and low birth weight rate in our study was lower compared with those observed by others and this may be derived from the benefit of using single blastocyst transfer in our study, which can markedly reduce the risk of multiple pregnancy and other associated complications.

In this study, the LAS group showed significantly increased blastocyst implantation rate, but this was also linked with a significantly increased monozygotic twin rate. Monozygotic twins are associated with a range of well-documented risks associated with the health of the mother and fetus. The rate of monozygotic twinning in the population in thought to be fairly low. However, the rate is increased in assisted reproductive technology [24, 25]. Some research showed that the incidence of monozygotic twinning may be affected by micro-manipulating the zona pellucida, in such procedures as ICSI and assisted hatching [26, 27]. Thus the rate of monozygotic twinning increased in the LAS group and this may be associated with the damage of the zona pellucida, creating a hole greater than the micro-needle puncture.

## Conclusions

Our results showed that LAS and MNAS of blastocoele prior to vitrification can both achieve favorable clinical and neonatal outcomes. Despite no differences in neonatal outcome, LAS appears to improve blastocyst implantation rate and live birth rate significantly compared with MNAS, but there is also an increased risk of producing monozygotic twins. Therefore, from this study we cannot make a conclusive decision on which method is better. For a more definitive conclusion and to assess the safety of artificial shrinkage a prospective randomized trial is needed.

## Abbreviations

ART: Assisted reproductive technology
DMSO: Dimethyl sulfoxide
FET: Frozen embryo transfer
HAS: Human serum albumin
HCG: Human chorionic gonadotropin
ICM: Inner cell mass
ICSI: Intracytoplasmic sperm injection
LAS: Laser pulse artificial shrinking
MNAS: Micro-needle artificial shrinking
TS: Thawing solution
